# Retrospective Study of Healthcare Resources Developed for Patients by Interprofessional Teams

**DOI:** 10.7759/cureus.33840

**Published:** 2023-01-16

**Authors:** Rebecca White, Ashley Walczybock, Jennifer Mendez, Ashley Reed, Shannon Maloney, Michelle Malik, Christine Kivlen

**Affiliations:** 1 Medicine, Wayne State University School of Medicine, Detroit, USA; 2 Health Care Sciences, Occupational Therapy, Wayne State University, Detroit, USA; 3 Kinesiology, Health, and Sports Studies, Wayne State University, Detroit, USA

**Keywords:** interprofessional team, older adults, medical education curriculum, covid-19, chronic condition

## Abstract

Wayne State University (WSU) emphasizes the importance of interdisciplinary education by having students participate in an Interprofessional Team Visit (IPTV) program. A 60-minute virtual visit is conducted to assess adults aged over 50 years within the Detroit Metropolitan Area (Metro Detroit) community. This project was designed to prepare healthcare students in evaluating the mental, physical, and social health aspects of assigned patients based on specific disciplinary assessments. Upon completion of assessments, the interdisciplinary team provided the patient with resources based on the team and the patient's agreed-upon area of concern. Twenty-eight IPTV teams, consisting of a medical and occupational therapy student and a healthcare professional student from another discipline studying at WSU, were randomly created. The IPTV resource guides created by each team were reviewed and sorted into two categories based on the health or social need of the individual patient. The data identified three main areas of interest, which included medication management, diet and exercise plans, and the use of technology to stay connected to medical professionals, friends, and family. The purpose of this report is to assess the IPTV program’s findings and analyze patients’ concerns based on health or social needs and the resources presented to them.

## Introduction

Current published research consistently reiterates the value of interprofessional experiences (IPE), not only to students during their education but also in everyday practice. Conti et al. discussed the ability to increase team functioning based on a health professional’s ability to understand and explain a variety of topics within healthcare knowledge [1]. Studies based on IPE also identified improvements in communication skills among healthcare professionals, with respect to making appropriate referrals [2]. This helps medical professionals value collaboration when addressing the health and social needs of a patient.

Early exposure to interprofessional team collaboration can be beneficial for students pursuing a health science career. Schiller et al. analyzed the perceptions of students in the Doctor of Physical Therapy (DPT) program at Wayne State University (WSU), who participated in an Interprofessional Team Visit (IPTV) program with older adults [3]. It was concluded that the DPT students benefitted primarily in communication skills, teamwork, and gaining general knowledge about other health professional fields. Similarly, Liaw et al. discussed how a three-dimensional virtual environment (3D-VE) enhances healthcare students' experience with collaborative learning [4]. In the study, six interprofessional teams were formed and students displayed improved attitudes toward healthcare teams and interprofessional collaboration. Students felt “immersed in their own roles as the virtual environment was perceived as ‘less threatening’ compared to face-to-face interactions,” [4]. Later analyses emphasized how students better understood the roles of other healthcare professionals in the health and wellness of a patient when multiple disciplines are included in a team [5].

The coronavirus disease 2019 (COVID-19) pandemic presented a new challenge for older adults in heavily impacted communities to continue seeking appropriate care for a health or social need. WSU saw this as an opportunity to integrate interprofessional team exposure with general practice skills when caring for aging individuals.

Background

Implementation of the Patient Protection and Affordable Care Act and shortages in the workforce have ampliﬁed the need for collaboration and teamwork across health professions [6]. To address this recent change, WSU implemented an IPTV with older adults living in the Detroit Metropolitan Area (Metro Detroit) community [7]. The IPTV program assessed adults aged over 50 years within the community. During the 60-minute virtual visit, each IPTV team gathered information regarding the adult’s daily activities, nutrition, medications, family health, and social support system. No other health information was collected. Next, the team members collaborated to determine the individual's current health status, identify areas of concern, and provide support and/or resources based on an agreed-upon need for the patient. This project focused on issues related to health and social needs amidst COVID-19.

Portions of this article were presented as a poster at the Nexus Summit 2021 on September 14, 2021

## Materials and methods

Participants

For the purpose of this study, a convenient sample of WSU students in the medical school, occupational therapy program, and a third healthcare field of study was randomly pre-assigned into 28 groups. The healthcare professional students for the third category were selected from one of the following WSU programs or a WSU-affiliated school/program: nursing, pharmacy, physical therapy, physician assistant, dental, or social work.

The patients were recruited through public health screenings at the Detroit RiverWalkers Program [8] and included other willing adult participants from the community. Inclusion criteria for participants included being at least 50 years old. Patient ages ranged from 50-89; 3.6% of patients were aged 50-59, 28.6% of patients were aged 60-69, 53.6% of patients were aged 70-79, and 14.3% of patients were aged 80-89. The gender proportion was not collected at that time.

No racial or ethnic exclusion criteria were included in the study; thus, the study consisted of White (75%), African American (21.4%), and Asian (3.6%) populations. The number of medications being taken by patients was also taken into consideration during the recruitment process, as pharmacy students conducted a medication tally assessment. Of the patients, 17.9% took zero to one medication, 57.1% of patients took two to three medications, and 25% of patients take four or more medications.

IPTV project data collection

Data collection occurred 13 months prior to the initiation of this retrospective study. IPTV groups collaborated with their assigned older adult to schedule a meeting via Zoom (Zoom Video Communications, Inc., San Jose, California, United States) or Microsoft Teams (Microsoft Corporation, Redmond, Washington, United States). Virtual visits were chosen as they have been shown to be an effective communication tool in education and medicine, as well as the added benefit of keeping patients and students safe during the COVID-19 pandemic [9-12]. Students performed assessments in real time during patient interviews on information based on prior health conditions and new concerns stemming from the COVID-19 pandemic. It is from these assessments and interview questions that students crafted their resource support projects to send to their patients.

The protocol for the students on the IPTV team was as described: students were required to meet with their older adult patient within 72 hours of the assignment. After the meeting, students collaborated with their interdisciplinary team to create a resource support project. This was due no later than two weeks after the orientation to the IPTV course. Submission reminder emails were sent to the entire class regardless of submission status at two weeks, one week, and one day prior to the submission deadline. Students were instructed to use scholarly resources when applicable. For example, if a patient requested resources pertaining to COVID-19, students would provide them with the updated CDC recommendations.

Research design

A qualitative study design was utilized for this research study to perform a retrospective examination of 28 IPTV resource guides that were provided to each patient. IPTV resource guides were reviewed by researchers to identify patient needs. Patient needs were classified as “health,” “social”, or “both.” For example, a health need by Patient A was “management of osteoporosis” and a social need by Patient B was “keeping in touch with friends.” A patient’s needs would have been classified as “both” if they requested assistance with, for example, hypertension (health) and insurance payments (social). Categorization of patient needs was first done individually among six researchers. Next, the research team as a whole reviewed their individual findings to identify the main themes in the data collected. Overall, 10 categories were created based on patient responses (Table 1).

**Table 1:** Patient Response Categories Patient concerns were sorted into one of 10 categories as determined by the researchers. The total number of each category is displayed in the bottom row. Categories 5 and 10 were considered "social" needs, the rest were considered "health" needs. COVID-19: coronavirus disease 2019

Pt	Concerns	Category: Weight Management	Category: Chronic Condition	Category: Exercise	Category: Smoking	Category: Safe Social Engagement Due to COVID-19	Category: Mental Health	Category: Nutrition	Category: Sleep	Category: Acute Condition	Category: Health Insurance
1	1) weight management 2) osteoporosis	X	X								
2	1) weight management	X									
3	1) hypertension		X								
4	1) exercise			X							
5	1) smoking				X						
6	1) weight management 2) gout	X	X								
7	1) weight management 2) safe social engagement 3) maintaining positive mental health	X				X	X				
8	1) high cholesterol 2) exercise 3) safe social engagement		X	X		X					
9	1) arthritis 2) exercise		X	X							
10	1) exercise 2) proper nutrition			X				X			
11	1) exercise 2) proper nutrition			X				X			
12	1) improving sleep								X		
13	1) weight management 2) exercise 3) proper nutrition	X		X				X			
14	1) high cholesterol 2) proper nutrition		X					X			
15	1) management of new-onset Guillain-Barre syndrome									X	
16	1) exercise 2) proper nutrition			X				X			
17	1) osteoporosis		X								
18	1) weight management 2) sleep 3) neck pain from recent accident	X							X	X	
19	1) payment of health insurance										X
20	1) safe social engagement					X					
21	1) exercise			X							
22	1) safe social engagement					X					
23	1) safe social engagement					X					
24	1) weight management	X									
25	1) exercise			X							
26	1) new back pain due to recent accident									X	
27	1) weight management	X									
28	1) pain due to recent hand trauma									X	
	TOTAL	8	7	9	1	5	1	5	2	4	1

The resource guides themselves were also analyzed. Research team members counted the number of interdisciplinary avenues of assistance listed in the 28 guides. For example, a patient with osteoporosis of the hip was offered a consultation for a hip replacement, the addition of vitamin D and alendronate to her medications, and home physical therapy to increase her strength. This would be considered “three” resources the team was able to provide to their patient.

Further resource guide analysis included the type of resources offered to a patient. Using the same methods as those used to categorize patient needs, resources were classified into the three categories: clinical care, at-home remedies, and improving access to healthy living. For example, a clinical care resource provided to Patient C was adding a medication to a current regimen; an at-home remedy provided to Patient D was home exercises for neck pain; and a resource to improve access to healthy living provided to Patient E was information on the city’s insurance assistance program.

Data analysis required both quantitative and qualitative methods to thoroughly reach conclusions. Quantitative methods produced descriptive statistics of the categories formulated based on overall patient needs. Qualitative data analysis stemmed from Strauss and Corbin [13] and occurred once quantitative analysis was complete. Initial data analysis involved open coding based on the area of concern chosen by IPTV team for their individual patient, which revealed specific health and social needs to create overall themes. Axial coding was then used to reveal similarities and differences amongst the themes generated from the open coding.

## Results

Initial results of the IPTV program

Of all the older adult volunteers, 100% responded to students when contacted about the virtual visit. Paralleling this, 100% of IPTV teams submitted a resource project. In total, 28 projects were submitted. Within these projects, students addressed one to two needs based on the patients' results from the various assessments and interview questions.

Results from the retrospective analysis

Table 1 describes the distribution of data for the retrospective study. Qualitative data demonstrated 78.6% of patients requested a "health” need, 14.3% of patients requested a “social” need, and 7.2% of patients requested “both”. Qualitative themes derived from patient responses included CDC-recommended guidelines, weight management, and how to safely interact with others during a pandemic. Of the 10 decided-upon categories shown in Table 1, five main themes of patient needs emerged, which included increased exercise (nine responses), weight management (eight responses), management of chronic conditions (seven responses), proper nutrition (five responses), and safe social engagement during a pandemic (five responses).

Table 2 describes the number and distribution of different types of resources offered to the patients. The number of resources offered ranged from one to six per patient. 25% (seven of 28) of guides offered only one resource, 35.7% (10 of 28) of guides offered two resources, and 39.3% of guides offered three or more resources. The total number of resources provided across all patients was 68; thus, on average, each patient was offered 2.4 different interdisciplinary resources to help them tackle their health or social need.

**Table 2:** Categorization and Listing of Resources Provided to Patients List of the resources provided for each patient, as sorted into one of three categories. Each resource is delineated by a bullet point. The total number in each category of resource is shown at the end of the row, and the total number of all resources is in the bottom right corner. USPSTF: United States Preventative Services Task Force; DEXA: dual energy x-ray absorptiometry; OT: occupational therapy; NIH: National Institutes of Health; UCSF: University of California San Francisco; PT: physical therapy

Patient #	1	2	3	4	5	6	7	8	9	10	11	12	13	14	15	16	17	18	19	20	21	22	23	24	25	26	27	28	Total
Clinical Care Resource		•CDC recommended vaccine list •CDC guidelines for pap smear •CDC recommendation for Calcium and Vitamin D in diet	•CDC guidelines for living with Hepatitis C •USPSTF DEXA recommendations •OT referral			•CDC recommendation for Calcium and Vitamin D in diet •USPSTF Screening Recommendations •Ophthalmology Consult for Dry Eyes		•CDC recommendations for Calcium and Vitamin D in diet •USPSTF DEXA recommendation							•Sleep foundation recommendation for melatonin dosing	•Mayo Clinic recommendations for inhaler for shortness of breath		•Ophthalmology referral	•Link to purchase glucose monitor •NIH website for stroke prevention				•CDC recommendations for Calcium and Vitamin D in diet	•CDC information for knee-replacement surgery					18
At-Home Remedy	•App to help track diet •Yoga app •CDC weight loss info •Exercise videos on YouTube •Exercise app •List of recipes found online	•Exercise diagram •WHO nutritional pamphlet	•CDC and FDA smoking cessation pamphlets	•Online recipe link •YouTube recipe videos	•CDC sleep guidelines and pamphlet		•CDC Smoking Cessation Pamphlet	•List of protein powder supplements from local grocery store •CDC list of exercises to reduce falls	•UCSF Sports Medicine recommended exercises			•American Heart Association dietary pamphlet •American Heart Association links for at home health improvement	•YouTube exercise videos •Links to online recipes	•YouTube exercise videos	•Sleep foundation recommendation for sleep hygiene •YouTube exercise videos •YouTube stretching videos	•YouTube and Silver Sneakers organization exercise videos	•YouTube exercise videos •Guillain-Barree online support group	•Mayo Clinic DASH diet recommendations •Online recipes to avoid Gout		•CDC recommended exercise to improve cardiovascular health	•CDC covid guidelines for staying safe at home	•Online PT exercises for hammer toe pain	•YouTube exercise videos •CDC DASH diet recommendations	•Healthline website link for exercise for back pain •Healthline website link for dietary planning	•CDC recommendations for at home isolation	•American Heart Association DASH diet plan •YouTube exercise videos	•Mindfulness podcast •Fitness App •Link to Detroit-area volunteer activities	•Link to purchase ergonomic posturing seat •YouTube exercises	44
Improving Access to Healthy Living										•Links to local library •links to books recommended by local library	•State government website links for insurance coverage •Links to Inexpensive medication •Links to free clinics in Detroit														•CDC recommendations for receiving care during COVID				6
																													68

Regarding the resources themselves, 26.5% offered involved clinical care, 64.7% offered at-home remedies, and 8.8% offered improved access to healthy living. Table 2 summarizes these findings, with the resource(s) provided to each patient sorted into the aforementioned categories. CDC recommendations made up the bulk of resource sources at 26.5%. As previously mentioned, students were instructed to use scholarly resources (CDC, United States Preventative Services Task Force (USPSTF), American Heart Association, etc.) when creating their resource documents, but 23.5% of resources involved a user-friendly site such as a recipe blog or a YouTube (Google LLC, Menlo Park, California, United States) exercise video. This was deemed more appropriate in these examples as it benefits the patient to have access to audio and visual demonstrations of potentially complex exercise or cooking techniques.

## Discussion

The role of IPTV is to promote interprofessional collaboration at the early stages of a healthcare professional student’s curriculum. This in turn helps the student to become confident in their clinical skills and improves communication skills between various healthcare professionals and patients. WSU’s IPTV program provides the opportunity to help the Metro Detroit community by allowing healthcare students to evaluate various aspects of a patient’s mental, physical, and social health through specific disciplinary assessments.

This retrospective study focused on the significant impact that early interprofessional collaboration had on health science students. It also provided insight as to how the IPTV program is run at WSU and the positive influence it has not only on students but older adults living in the Metro Detroit community. Following IPTV virtual visits, the IPTV teams developed resource guides that addressed either “health”, “social”, or “both” needs. The IPTV resource guides provided useful information for patients, helping improve their quality of life; resources ranged from clinical care to at-home remedies, or to make healthy living more accessible. It also proves to students the importance of considering interprofessional care for their future patients as a way to increase healthcare quality as students used their experiences in their respective healthcare fields to craft holistic resource guides.

Patient responses allowed researchers to identify and classify main themes based on qualitative data, such as increased exercise, weight management, chronic condition management, proper nutrition, and social engagement. Of these common themes, we have been able to reference other research to support the need for such resources for older adults. Forde et al. explained in Ireland, there was an increase in adults participating in physical activity during the pandemic [14]. This was due to the increased available time to exercise or the belief that exercising was important and the ease of access to home-based and online resources [14]. Weight management proved to be difficult, as loneliness and working remotely decreased behaviors of staying active, planning and tracking food consumption, choosing healthy foods, and reducing emotional eating [15]. Freedman shed light on how older adults’ social contact changed during the pandemic as well [16]. He explained that weekly in-person contact dropped significantly for those in residential care; however, adults with electronic, telephone, or video contacts exhibited increased weekly or more contact. The findings provided by Freedman supported how communication technologies are important for older adults to maintain social ties, just as IPTV teams provided resources on how to access social engagement safely. 

As for patient care, health professional students were able to receive a greater understanding of collaboration with various disciplines, interacting with patients through telehealth, identifying areas of concern for patients, and providing the necessary resources and tools to help. Similar findings were also identified by Conti et al. acknowledging that IPE allows students to increase their awareness of the importance of interprofessional collaboration in relation to patient care [1].

Limitations

Virtual IPTV visits were conducted using formats such as Zoom or Microsoft Teams. This proved difficult for some groups as there were technical issues related to sound and video communication as well as patient difficulty navigating these online resources. Instructions could be provided in the future on what common issues are encountered when using telehealth and how to quickly troubleshoot them [17]. Further research could be done to explore health and/or social needs within a more specific patient population. This could allow researchers to identify common themes found based on a narrower patient population to better identify problem areas earlier on in their health journey. Also, students would be able to test their current knowledge of a specific population and increase collaboration with interdisciplinary team members.

## Conclusions

The aim of this report was to focus on an expressed health or social need by a patient and the type of resources provided to the patient. These findings suggest that a student-based interdisciplinary team collaboration allows for full consideration of the patient's mental, physical, and social health concerns. Overall, resources that were provided consisted mostly of health needs, suggesting limited access to medical resources due to the pandemic. Patient concerns based on social needs emphasized the value of face-to-face interactions on a daily basis.

The interprofessional team collaborated to improve patients’ health and social outcomes. This program provided students with virtual opportunities to recognize patient needs, acquire resources, and work with individuals aged over 50 years to improve their quality of life. The impact of COVID-19 forced students to think critically about social determinants of health as well as what needs and resources were feasible for each patient.

## References

[1] Conti G, Bowers C, O'Connell MB (2016). Examining the effects of an experiential interprofessional education activity with older adults. J Interprof Care.

[2] Khan AI, Barnsley J, Harris JK, Wodchis WP (2022). Examining the extent and factors associated with interprofessional teamwork in primary care settings. J Interprof Care.

[3] Schiller M, Gilkey S, Mendez J, Dunleavy K (2019). An interprofessional team experience—value and timing in a doctor of physical therapy curriculum. Journal of Physical Therapy Education.

[4] Liaw SY, Soh SL, Tan KK (2019). Design and evaluation of a 3D virtual environment for collaborative learning in interprofessional team care delivery. Nurse Educ Today.

[5] Davis J, Zulkosky K, Ruth-Sahd LA, Frank EM, Dommel L, Minchhoff D, Uhrich K (2021). Health care professional students' perceptions of teamwork and roles after an interprofessional critical care simulation. Dimens Crit Care Nurs.

[6] Zhang X, Lin D, Pforsich H, Lin VW (2020). Physician workforce in the United States of America: forecasting nationwide shortages. Hum Resour Health.

[7] Sedki M, Mendez J, Bruer S, Levine D (2015). The importance of teamwork in healthcare for effective communicate and care of older adults. J Interprof Educ Pract.

[8] (2022). Detroit Riverwalkers. https://detroitriverfront.org/2022detroitriverwalkers.

[9] Weinstein RS, Lopez AM, Joseph BA, Erps KA, Holcomb M, Barker GP, Krupinski EA (2014). Telemedicine, telehealth, and mobile health applications that work: opportunities and barriers. Am J Med.

[10] Dulebohn JH, Hoch JE (2017). Virtual teams in organizations. Hum Resour Manag Rev.

[11] Rubinger L, Gazendam A, Ekhtiari S (2020). Maximizing virtual meetings and conferences: a review of best practices. Int Orthop.

[12] Correia AP, Liu C, Xu Xu, F F (2020). Evaluating videoconferencing systems for the quality of the educational experience. Distance Educ.

[13] Corbin J, Strauss A (2014). Basics of Qualitative Research: Techniques and Procedures for Developing Grounded Theory. https://us.sagepub.com/en-us/nam/basics-of-qualitative-research/book235578.

[14] Forde C, Wyse J, Barrett E (2022). Time and belief in exercise importance predict increased activity during initial COVID-19 restrictions in Ireland. Health Promot Int.

[15] Borgatti AC, Schneider-Worthington CR, Stager LM (2021). The COVID-19 pandemic and weight management: effective behaviors and pandemic-specific risk factors. Obes Res Clin Pract.

[16] Freedman VA, Hu M, Kasper JD (2022). Changes in older adults' social contact during the COVID-19 pandemic. J Gerontol B Psychol Sci Soc Sci.

[17] (2022). Common Videoconferencing Problems and How to Fix Them. https://fedtechmagazine.com/article/2020/04/common-videoconferencing-problems-and-how-fix-them-perfcon.

